# Viability-PCR Allows Monitoring Yeast Population Dynamics in Mixed Fermentations Including Viable but Non-Culturable Yeasts

**DOI:** 10.3390/foods9101373

**Published:** 2020-09-27

**Authors:** Yurena Navarro, María-Jesús Torija, Albert Mas, Gemma Beltran

**Affiliations:** Department of Biochemistry and Biotechnology, Faculty of Oenology, University Rovira i Virgili (URV), Marcel·lí Domingo 1, 43007 Tarragona, Catalonia, Spain; yurenadelosangeles.navarro@urv.cat (Y.N.); albert.mas@urv.cat (A.M.); gemma.beltran@urv.cat (G.B.)

**Keywords:** non-*Saccharomyces*, *Saccharomyces cerevisiae*, wine yeast, viable but non culturable, viability qPCR

## Abstract

The use of controlled mixed inocula of *Saccharomyces cerevisiae* and non-*Saccharomyces* yeasts is a common practice in winemaking, with *Torulaspora delbrueckii*, *Lachancea thermotolerans* and *Metschnikowia pulcherrima* being the most commonly used non-*Saccharomyces* species. Although *S. cerevisiae* is usually the dominant yeast at the end of mixed fermentations, some non-*Saccharomyces* species are also able to reach the late stages; such species may not grow in culture media, which is a status known as viable but non-culturable (VBNC). Thus, an accurate methodology to properly monitor viable yeast population dynamics during alcoholic fermentation is required to understand microbial interactions and the contribution of each species to the final product. Quantitative PCR (qPCR) has been found to be a good and sensitive method for determining the identity of the cell population, but it cannot distinguish the DNA from living and dead cells, which can overestimate the final population results. To address this shortcoming, viability dyes can be used to avoid the amplification and, therefore, the quantification of DNA from non-viable cells. In this study, we validated the use of PMAxx dye (an optimized version of propidium monoazide (PMA) dye) coupled with qPCR (PMAxx-qPCR), as a tool to monitor the viable population dynamics of the most common yeast species used in wine mixed fermentations (*S. cerevisiae*, *T. delbrueckii*, *L. thermotolerans* and *M. pulcherrima*), comparing the results with non-dyed qPCR and colony counting on differential medium. Our results showed that the PMAxx-qPCR assay used in this study is a reliable, specific and fast method for quantifying these four yeast species during the alcoholic fermentation process, being able to distinguish between living and dead yeast populations. Moreover, the entry into VBNC status was observed for the first time in *L. thermotolerans* and *S. cerevisiae* during alcoholic fermentation. Further studies are needed to unravel which compounds trigger this VBNC state during alcoholic fermentation in these species, which would help to better understand yeast interactions.

## 1. Introduction

Alcoholic fermentation of grape must is mainly driven by *Saccharomyces cerevisiae*, which quickly dominates the fermentation of grape must in wine production. However, in the last years, there has been an increasing interest in studying other fermentative yeasts, which are generally referred to as non-*Saccharomyces* yeasts, due to the ability of some of them to improve the complexity of wines by increasing the concentration of aromatic molecules, such as terpenoids, esters, higher alcohols or other molecules of interest, such as glycerol [1,2,3,4,5,6]. In addition, another advantage is the potential of some species to reduce the alcohol content of wines, a feature increasingly sought after in this industry [7,8,9]. In contrast, some of these non-*Saccharomyces* yeasts are known to spoil wines and cause stuck fermentations [10,11]. However, several studies have shown that when selected non-*Saccharomyces* yeasts are used as starters together with *S. cerevisiae*, they are able to participate in the fermentations until late stages, and produce wines with the previously mentioned characteristics [3,12]. Among them, it is important to highlight those species that are already available as commercial strains, *Metschnikowia pulcherrima*, *Torulaspora delbrueckii* and *Lachancea thermotolerans*. All of them showed the ability to reduce ethanol content, mainly *M. pulcherrima* [9,13,14,15,16,17]. In addition, *M. pulcherrima* has been described as a yeast that makes wines fruitier and fresher, positively modulating the wine aroma profile [13,18]. *L. thermotolerans* increases wine natural acidity due to increased lactic acid production [13,19,20] and *T. delbrueckii* has a positive influence on the overall impression of the obtained wines, improving the aroma quality and the varietal character [19,21,22].

Therefore, although it has been proved that the use of some of these yeasts together with *S. cerevisiae* as starters produces wines with improved qualities, their interactions at the microbiological level are still not completely understood. Very early studies of alcoholic fermentation confirmed that *S. cerevisiae* quickly dominates the fermentation process, resulting in the disappearance of the non-*Saccharomyces* yeasts, which was initially attributed to different ethanol and SO_2_ sensitivities of the latter [23]. However, different studies during the last 20 years have demonstrated the ability of many non-*Saccharomyces* yeasts to survive until the end of the fermentation, with a broad array of interactions among different yeasts [2,3,12]. Indeed, the yeast interactions are highly dependent on the species and strains used [24,25]. From the beginning of the fermentation, there is competition for nutrients (mainly for nitrogen) and oxygen, which can influence the behavior of these yeasts and, consequently, the final wine. During alcoholic fermentation, some studies have demonstrated that the secretion of antimicrobial peptides by *S. cerevisiae* is responsible for the premature death of non-*Saccharomyces* yeasts [26,27,28]. Furthermore, direct physical contact between non-*Saccharomyces* and *S. cerevisiae* yeasts can also cause different responses in the cells. In fact, several studies have shown early growth arrest of different non-*Saccharomyces* yeasts in co-fermentations with *S. cerevisiae* due to a cell–cell contact mechanism [29,30,31,32].

The detection and quantification of the yeasts involved in wine production have been studied for many years and with different methodologies. The most widely used method is based on the culturability on different solid media. The use of differential media, such as Wallerstein Laboratory Nutrient (WLN) medium, allows the differentiation of some non-*Saccharomyces* yeasts, but not all can be differentiated with this method, as not all the species present different colony morphologies [33]. Indeed, culture-dependent methods are time-consuming and unable to detect viable but non-culturable (VNBC) microorganisms, and even some slow-growing microorganisms may also be undetected. Thus, to achieve an accurate study of population dynamics, it is important to use culture-independent techniques, which will allow the identification and quantification of the different yeasts present during alcoholic fermentation regardless of their culturability. Several molecular tools have been developed, with quantitative polymerase chain reaction (qPCR) being one of the most applied methods [34,35,36,37]. Several studies have demonstrated that qPCR is much more specific than other molecular tools in the detection of different yeast and bacteria during fermentation [38,39]. In addition, qPCR has been shown to be sensitive enough to detect even underrepresented strains among not only yeasts [35,40,41], but also other types of wine microorganisms, such as bacteria [42,43].

As mentioned before, interactions among yeasts during alcoholic fermentation can lead to cell death or growth arrest. If this occurs, the qPCR results will not be accurate enough since the test quantifies the total DNA, not differentiating between dead and living yeasts. To solve this problem, some studies have applied reverse-transcription PCR (RT-PCR), a technique that amplifies the genetic material only from viable cells [34,44,45]. However, as RNA handling is more demanding and prone to degradation by contamination with RNA-degrading enzymes, this tool requires trained personnel and may result in problems of reproducibility. A simpler procedure is the use of viability-qPCR, which includes a pretreatment of the sample with DNA-binding dyes, such as ethidium monoazide (EMA) or propidium monoazide (PMA). These dyes enter cells with damaged membranes and bind to DNA in a covalent manner after photoactivation, preventing its amplification by subsequent PCR. This means that only DNA from viable cells (with membrane integrity) will be susceptible to being amplified by qPCR [38,46,47,48,49]. Several studies performed in bacteria and parasites showed that PMA is an excellent choice to determine the viability of cells due to its high specificity and sensitivity in different food matrices, such as oysters, meat and wastewater [50,51,52]. Indeed, PMA seemed to be more selective for dead cells than EMA [53,54]. A new and improved version of PMA, PMAxx, has been shown to be better at discriminating between living and dead bacterial cells or viruses [55,56,57]. However, only a few studies have been conducted using viability-qPCR to determine yeast population dynamics through alcoholic fermentation [38,44,45,58].

Our aim in this study was to analyze the population dynamics of mixed alcoholic fermentation without the use of SO_2_ in order to eliminate competitive advantages for *S. cerevisiae*, and to determine the VBNC population. For that reason, we simultaneously inoculated a synthetic must with *S. cerevisiae* and three non-*Saccharomyces* yeasts commonly used in mixed wine industrial fermentations (*T. delbrueckii*, *L. thermotolerans* and *M. pulcherrima*), and analyzed yeast dynamics and viability for each species by PMAxx-qPCR and compared the results with those from non-dyed qPCR and colony counting.

## 2. Materials and Methods

### 2.1. Strains and Culture Conditions

Four yeast species were used in this study: *S. cerevisiae* QA23 (Lallemand Inc., Montreal, QC, Canada) (Sc), *T. delbrueckii* Biodiva (Lallemand Inc., Canada) (Td), *L. thermotolerans* 1 (provided by Agrovin S.A., Alcázar de San Juan, Spain) (Lt) and *M. pulcherrima* CECT 13131 (Mp) isolated from grape must from the Priorat region (URV collection) [25]. Strains were plated on YPD solid medium (2% (*w*/*v*) glucose, 2% (*w*/*v*) yeast extract, 1% (*w*/*v*) peptone and 1.7% (*w*/*v*) agar) and on Wallerstein Laboratory Nutrient (WLN) agar (Becton, Dickinson and Company, Le Point de Claix, France) from frozen stocks, and one colony was used to develop the inoculum. Before starting fermentations, routine species confirmation of the different strains was performed by restriction analysis (PCR-RFLP) of 5.8 S-ITS rDNA [59].

### 2.2. Fermentation Procedure

Single colonies of each strain were grown individually in YPD liquid medium for 24 h at 28 °C. Cells were counted in a Neubauer chamber and 5 × 10^5^ cells/mL of each strain were simultaneously inoculated in 230 mL of a synthetic must [60], contained in 250 mL bottles. Thus, the total inoculated population was 2 × 10^6^ cells/mL. Fermentations were performed in triplicate and incubation was at 22 °C with stirring (120 rpm).

Fermentations were monitored by measuring must density (electronic densitometer, Densito 30PX Portable Density Meter; Mettler Toledo, Spain) and optical density at 600 nm (Ultrospec 2100 Pro; Biotech Ltd., Cambridge, England). Fermentations were considered to be finished when residual sugars were determined to be less than 5 g/L (Miura One Rev; I.S.E. Srl, Italy).

### 2.3. Population Dynamics by Plating

The population dynamics were monitored by plating samples at different points of fermentation on two solid media. Briefly, samples were serially diluted in sterile water and the number of colony-forming units per milliliter (CFU/mL) was determined by plating 100 μL of the corresponding dilutions on YPD for total yeast population and on WLN for the morphological differentiation and counting of the four species present in the sample.

### 2.4. Population Dynamics by qPCR

#### 2.4.1. Living/Dead Cell Amplification: Heat Shock and PMAxx Treatment

To better differentiate between living and dead yeast cells and to ensure that the qPCR only amplifies living cells, the PMAxx^TM^ viability dye (Biotium Inc., Fremont, CA, USA) was used. First, we validated the effectiveness of this dye using living cells and heat-treated cells (as dead cells).

One yeast colony was inoculated in YPD liquid medium and incubated overnight at 28 °C. Then, two aliquots of 1 mL sample (10^6^ cells/mL) were centrifuged at 6000 rpm for 5 min and the pellet was resuspended in 1 mL of sterile distilled water. One of the aliquots was subjected to heat shock (95 °C for 10 min) and both were stained with PMAxx according to the manufacturer’s protocol, with the modification of the cross-link time. Briefly, the PMAxx dye (25 µM) was added to the sample, followed by an incubation time of 10 min in the dark. Then, based on previous studies [38], the sample was exposed twice to light for 30 s and to ice for 60 s in between light exposures. Cells, not treated with PMAxx or light exposure, were used as controls to evaluate the effect of the dyes. The pellet was recovered by centrifugation at 13,500 rpm for 2 min and DNA was extracted.

The effectiveness of heat shock treatment was confirmed by the absence of growth of the heat-treated cells on YPD solid medium compared with the growth observed for non-treated cells. The procedure was repeated at least twice with each species.

#### 2.4.2. PMAxx Treatment of Samples from Fermentation

Two 500 µL aliquots of must were centrifuged at 7800 rpm for 2 min and the pellets were washed with sterile distilled water. One aliquot was resuspended in 500 µL of sterile distilled water and then was treated with the PMAxx dye as previously described. The other aliquot was not treated (untreated cells). Then, DNA from both aliquots was extracted as described below.

#### 2.4.3. DNA Extraction and qPCR Analysis

DNA was extracted using the DNeasy Plant Mini Kit according to the manufacturer’s instructions (Qiagen, Hilden, Germany). Primers used to amplify non-*Saccharomyces* species were published by García et al. [36], *S. cerevisiae* primers were published by Hierro et al. [61] and the total yeast population was quantified using primers described by Hierro et al. [34] (Table A1). All qPCR amplifications were carried out in triplicate with a final volume of 20 µL using TB Green^TM^ Premix Ex Taq^TM^ II (Takara Bio Inc., Kusatsu, Japan) according to the manufacturer’s instructions in a QuantStudio^TM^ 5 real-time PCR instrument (Applied Biosystems by Thermo Fisher Scientific, Waltham, MA, USA). The amplification process included an initial denaturation at 95 °C for 30 s, followed by 40 cycles of denaturing at 95 °C for 5 s and annealing at 60 °C for 30 s. Cycle threshold (Ct) was determined using the Standard Curve application (Applied Biosystems by Thermo Fisher Scientific, Waltham, MA, USA). Primer specificity was checked by performing the same qPCR with DNA of all species involved in the study. Milli-Q water (Millipore, Molsheim, France) was used as a negative control of amplification (NTC).

For each species, standard curves, with and without PMAxx dye treatment, were created by plotting the average Ct values of a tenfold serial dilution of DNA from 10^7^ to 10 cells/mL against the log of cells/mL; and each dilution was assayed in triplicate. Standard curves were constructed using the Standard Curve application.

#### 2.4.4. Limit of Detection and Quantification by PMAxx-qPCR

The limit of detection (LoD) and quantification (LoQ) by qPCR of living cells treated with PMAxx were calculated in pure and mixed cultures. For the latter, two different types of combinations were prepared for each yeast species. In living/dead mixed cultures, tenfold serial dilutions of living cell DNA (from 10^4^ to 10 cells/mL) were mixed with DNA extracted from 10^6^ cells/mL of dead cells from the same species. Additionally, in living/living mixed cultures, the same serial dilutions of DNA from living cells were mixed with DNA extracted from 10^7^ cells/mL of living cells from another yeast species (Sc was mixed with Td; and Td, Lt and Mp were mixed with Sc). According to the Clinical and Laboratory Standards Institute (CLSI), the LoD is the lowest amount of analyte in a sample that can be detected with probability, although perhaps not quantified as an exact value (Ct < 40). The LoQ is the lowest amount of measurand in a sample that can be quantitatively determined with acceptable precision and stated, acceptable accuracy, under stated experimental conditions (Ct < 30).

### 2.5. Statistical Analysis

All analyses were performed using GraphPad Prism^®^ version 6 (GraphPad Software, San Diego, CA, USA). Results are expressed as the mean ± SD. Student’s *t*-tests were applied to analyze differences between living and dead cells and the influence of PMAxx treatment (*p* < 0.05).

## 3. Results

### 3.1. Optimization of PMAxx-qPCR for Yeast Viability Determination

**Figure 1:**
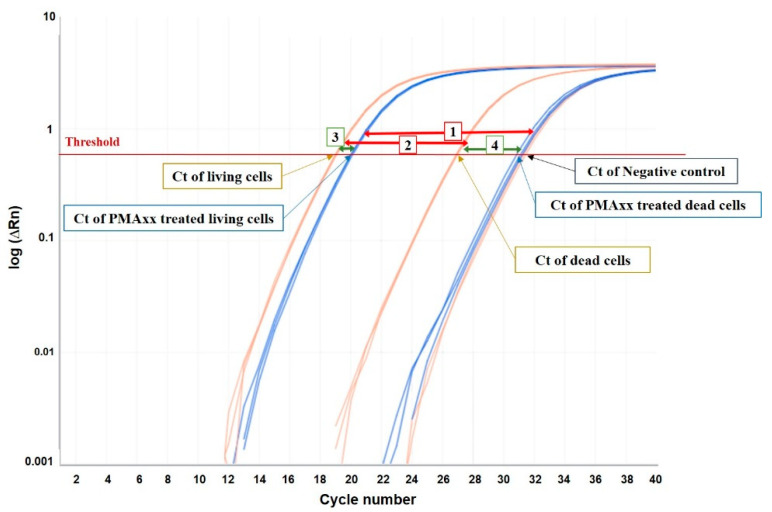
Schematic representation of the effect of PMAxx treatment in living and dead cells. Red arrows represent the cycle threshold (Ct) reduction obtained by subtracting the mean Ct values of living cells from those of dead cells, (1) with or (2) without PMAxx treatment. Green arrows represent the Ct reduction obtained by subtracting the mean Ct values obtained from PMAxx-qPCR of (3) living or (4) dead cells from the mean Ct values obtained from non-dyed qPCR.

PMAxx is an optimized version of PMA, a fluorescent photoaffinity dye that binds covalently to DNA after photoactivation, inhibiting its amplification by PCR. As PMAxx can only enter cells with compromised or damaged membranes, but not intact cells, predominantly, DNA from living cells will be detected by qPCR. In our study, dead cells treated with PMAxx showed the maximum possible Ct reduction (amplifying from 4.834 to 10.4 cycles later than untreated dead cells, depending on the yeast; Figure 1, Table 1a), as the Ct of these samples clustered with the negative controls (NTC) (Figure 1). Dead cells without PMA treatment amplified much earlier, but it was still later than the Ct of living cells. In contrast, living cells treated with PMAxx showed a small Ct reduction compared with untreated cells, being also dependent on the yeast (from 0.5525 to 2.171 cycles; Figure 1, Table 1a). Therefore, the impact of the treatment with the PMAxx dye on the DNA of living cells, although subtle, has to be considered in its quantification. Hence, when the means of Ct values from living and dead cells were compared (Figure 1, Table 1b), the PMAxx treatment resulted in a Ct reduction from 11.33 to 15.42 cycles (the maximum possible reduction), while the reduction in untreated cells showed significantly lower values (between 7.39 and 8.81 cycles).

Since the treatment with PMAxx affected the quantification of both living and dead cells, two standard curves were performed for each species to accurately quantify both treated and untreated samples—with and without PMAxx treatment of living cells. The correlation coefficients, slopes and efficiencies of the amplification of standard curves are shown in Table 2. Standard curves obtained with the different species had similar slopes, with efficiencies ranging between 97.1% and 108.7%, and with an *R*^2^ close or equal to 1 (Table 2). Indeed, we obtained better efficiencies (closer to 100%) with DNA from PMAxx-treated cells. The LoQ (Table 2) for all the species treated with PMAxx was 10^3^ cells/mL, which was linear over five orders of magnitude, except for Mp, which was 10^4^ cells/mL. Untreated cells of Sc and Td showed a lower LoQ (10^2^ cells/mL), while for Lt and Mp, no differences in LoQ were observed between PMAxx-treated and untreated cells. The LoD was one log lower in all cases (Table 2).

To determine if the presence of dead cells from the same species or living cells from other species modified the sensitivity of our detection and quantification, the LoD and LoQ of living cells treated with PMAxx were also calculated in mixed cultures. In all the species, both limits remained identical to those obtained in pure cultures (Table A2).

### 3.2. Monitoring Yeast Population Dynamics of Mixed Fermentation and Differentiation of VBNC Yeasts

Sc, Td, Lt and Mp were simultaneously inoculated in the synthetic must in the same proportion (1:1:1:1) to test if PMAxx-qPCR could be used to monitor the viable population dynamics of each species during mixed fermentation. The results of PMAxx-qPCR were compared with those from non-dyed qPCR and with a culture-dependent technique, using YPD and WLN differential media.

**Figure 2:**
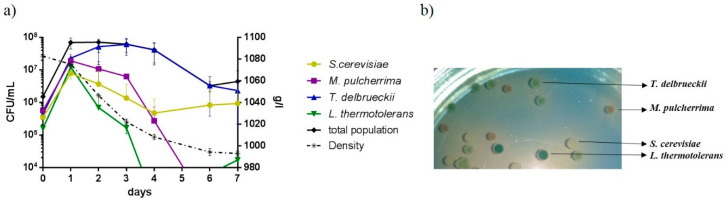
(**a**) Density (g/L) and viable population (CFU/mL) dynamics during fermentation. The total population was determined by plating on YPD medium and the viable population for each strain was determined by plating on WLN medium. The results are expressed as the mean ± SD of three fermentations. (**b**) Colony morphologies of the four species on WLN plates.

Mixed fermentations were completed in 7 days (Figure 2a). First, viability was analyzed using classical microbiological methods after plating the samples on YPD and WLN media. The four yeast species used in this study showed different colony morphology on WLN medium, allowing the counting of each strain separately on the same plate (Figure 2b). During fermentation and based on colony growth on WLN medium, at day 1, all species were able to grow and reached the same population (approximately 10^7^ CFU/mL), but since day 2, each species followed different dynamics. Td was the species with the highest number of CFU/mL counted on WLN plates, in all sampling points, with its maximum growth at day 3 (6.17 × 10^7^ CFU/mL). Sc colonies were detected throughout fermentation, but, surprisingly, Sc was not the main strain at the end of fermentation. In contrast, Mp and Lt colonies were not detected at all sampling points. Hence, Mp suffered a drastic fall in plate counts starting at day 3, so that the last colonies were observed at day 4. Lt was the species with the fastest decrease in plate viability, as no colony was observed after day 3; however, surprisingly, at day 7, some colonies were recovered in two out of three replicates. The absence of Lt colonies at days 4 and 6 showed that colony counting is not a suitable method for the detection of minority strains. The sample dilution necessary to have a proper number of colonies in a plate (30–300 CFU/mL) rules out the possibility of detecting low-abundance strains.

**Figure 3:**
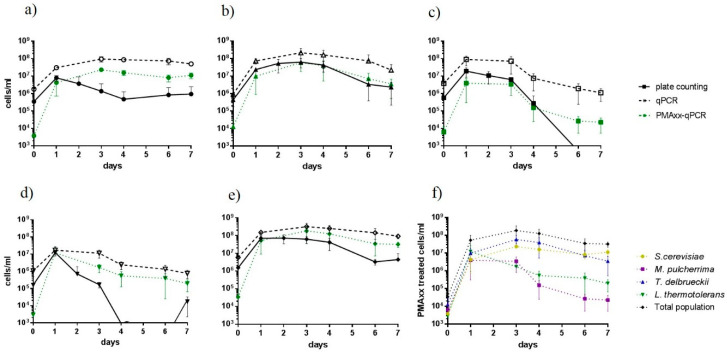
Yeast population analysis based on colony counting performed with WLN plates (black lines) and qPCR with or without PMAxx treatment (green dotted lines or black dashed lines, respectively) of (**a**) *S. cerevisiae*, (**b**) *T. delbrueckii*, (**c**) *M. pulcherrima*, (**d**), *L. thermotolerans* and (**e**) and total yeast population during fermentation. (**f**) Growth dynamics of the different yeast species based on PMAxx-qPCR results.

Plating results were then compared with qPCR analysis. For qPCR, samples of days 0 (1 h after inoculation), 1, 3, 4, 6 and 7, treated and untreated with PMAxx, were analyzed (Figure 3). In a general overview, for all yeast species, the results obtained with PMAxx-qPCR were closer to cell count, whereas non-dyed qPCR results showed population overestimation (Figure 3). Focusing on PMAxx-qPCR results, we observed that the populations estimated for Td and Mp species, as well as for total yeasts, were similar to those obtained by colony counting (except for the days with undetected Mp colonies on WLN medium). However, for Sc and Lt species, the culturable populations were lower than those determined by PMAxx-qPCR from day 3 to the end of fermentation. This difference was higher in Lt, reaching more than a 3-log unit difference at days 4 and 6. Therefore, if we observed the fermentation dynamics analyzed by PMAxx-qPCR (Figure 3f), expected results were obtained, with Sc being the main species detected at the end of the fermentation. Td presented a population similar to Sc, and both species (the ones with higher fermentative capacity) represented more than 98% of the yeast population detected at the end of fermentation. Interestingly, 1 h after inoculation, all yeasts showed a low viable cell number by PMAxx-qPCR, reaching up to two logarithm units less than colonies counted on plates.

## 4. Discussion

The study of microbial population in alcoholic fermentation has been traditionally performed by the classical microbiological colony-counting technique [4,23]. However, in the last 30 years, new methodologies have been applied because more accurate and specific techniques are needed to satisfy the demands of the winemaking industry. In our study, we aimed to validate PMAxx-qPCR as a tool to monitor the population dynamics throughout alcoholic fermentation of the most common yeast species (*S. cerevisiae*, *T. delbrueckii*, *L. thermotolerans* and *M. pulcherrima*) currently used in the wine industry, in comparison to non-dyed qPCR and classical microbiological methods.

The non-*Saccharomyces* species used in this study are all commercially available as active dry yeast starters and are used in mixed or sequential fermentations with *S. cerevisiae* due to the positive characteristics given to the final wine [14,16,18,19,62]. Thus, having a reliable methodology to analyze the population dynamics when using those species in mixed fermentations will be very useful to understand the survival of the different microorganisms and the imprint of the different species in the final product.

When analyzing the population dynamics of spontaneous or mixed fermentations, the classical solid medium used to study the cell population presents important drawbacks to properly distinguish and count all the species present [35,36], and further molecular analyses are needed to identify the different yeasts, usually by culture-dependent molecular techniques (RFLP-PCR of rDNA, rDNA sequencing, etc.) [59]. Some differential media such as WLN can overcome some of these problems since it is possible to distinguish and count different species based on colony morphologies [33]. However, not all species can be easily differentiated because some of them present similar aspects; additionally, colony morphology is strongly dependent on yeast physiological status. In our study, as mentioned above, yeast species were first selected based on their enological potential, but also because it was possible to differentiate the four strains on WLN medium, which allowed us to monitor them by colony counting. The WLN medium enabled us to determine the presence of the four yeast species until mid-fermentation (days 3 and 4). When the populations of some species (Mp and Lt) began to decrease, only few or no colonies could be detected at the chosen dilution. As all yeast species were detected in the same plate, the dilution rate had to be chosen depending on the total population, which allowed only the dominant species to be detected (species with populations 2 log units lower than the main species cannot be detected). Similar results have been observed in different studies [36,41,63], where the detection of minor strains could only be achieved after using qPCR. For these reasons, we consider that colony counting is not an accurate technique to follow the population dynamics when several yeasts are involved in fermentation, and at different cell populations, as they occur in natural habitats. Indeed, WLN medium can be used to detect and count species with similar log populations, but it cannot detect differences higher than 1 or 2 log units.

Opposite to colony counting, the use of qPCR allowed the detection of the four yeast species throughout the fermentation. In agreement with previous studies, the cell population numbers obtained by an independent-culture technique, such as qPCR, were higher than the results obtained by plating [36,38,39,61,63], and the difference between methodologies was higher as the fermentation progressed. This fact could be due to the inability of qPCR to differentiate DNA from living and dead cells. Therefore, as fermentation progresses, the number of dead cells increases, and, if the cell death rate is greater than the DNA degradation rate, the difference between colony counting and qPCR also increases.

To achieve an exact quantification of viable cells, samples were treated with PMAxx, so that only DNA from cells with an intact membrane, impermeable to this dye, can be amplified. Several studies have proven that this kind of dye, known as a viability dye, is useful to assume that the amplified population can be considered as viable cells [38,46,49,53,55]. In the case of studies of yeast viability performed by qPCR, only EMA and/or PMA have been used as viable dyes [38,44,45,58]; PMAxx has not yet been tested to determine its usefulness in yeast. The significant Ct reduction obtained between treated and untreated dead cells allowed us to validate the effectiveness of the treatment in all yeast species under study. Likewise, the Ct reduction between living and dead cells treated with PMAxx was significantly higher than that with untreated cells. However, a subtle Ct reduction was also obtained when living cells were treated with PMAxx. This fact exposed the need to calculate different standard curves depending on whether they are treated or not. According to results obtained by other authors [38,44], the quantification of a sample of treated living cells was not altered by the presence of DNA from dead cells of the same species (10^6^). In this study, the interference with DNA from different living cells (10^7^) was also tested, and again, linearity of standard curves was not influenced. In addition, LoD and LoQ were calculated in the presence of both exogenous DNA molecules and these limits were not changed in any condition.

The application of PMAxx-qPCR allowed us to obtain a more accurate determination of the population dynamics. At all sampling points analyzed, except for time 0, the quantification obtained by PMAxx-qPCR was closer to the results of colony counting, while non-dyed qPCR overestimated the population in all cases. As we have previously noted, this may be due to the ability of PMAxx to bind the DNA from dead cells, impairing its amplification, so that qPCR will mainly amplify and detect the DNA from viable cells [38,45,50]. Thus, at the sampling points where no species were detected by colony counting, PMAxx-qPCR results will be more reliable than qPCR ones since they are more accurate for viable cell counting. Similar conclusions were drawn by Vendrame et al. [45], but using PMA as a viability dye to assess wines spontaneously and artificially contaminated with *Brettanomyces bruxellensis,* obtaining better results by PMA-qPCR than by colony counting, qPCR or even RT-qPCR.

Nevertheless, some discordant points were detected. One of them was the low number of viable cells obtained by PMAxx-qPCR after one hour of must inoculation compared with colony counting or non-dyed qPCR. Capusoni et al. [64] showed how the permeability of the cell membrane of *S. cerevisiae* changes due to cultivation in a hyperosmotic medium (high NaCl concentration); this change allows the entry of several DNA-binding dyes into cells. Grape must is a hostile and stressful medium for yeast cells [65,66,67]. When yeast cells come into contact with grape juice, a sudden change in the expression of stress genes takes place, and *GPD1*, an osmotic stress gene, is activated within the first hour after inoculating grape juice from a stationary preculture [68]. In this way, this stress condition can also generate a change in the permeability of the cell membrane and therefore, allow the entry of PMAxx into viable cells, explaining the lower quantification after qPCR at this early time point.

However, the most important aspect revealed by the use of this viable dye was the existence of *S. cerevisiae* and *L. thermotolerans* in a VBNC state during fermentation. This behavior has been widely described by several authors in different bacteria and is considered as a survival strategy that permits resilience to unfavorable environmental conditions [50,69,70]. These cells retain an intact membrane, undamaged DNA and metabolic activity, but they are not culturable using laboratory media [71]. Some studies have also evidenced this phenomenon in wine yeasts [24,72] and explored the different stress conditions that could lead to the VBNC state in non-*Saccharomyces* yeasts. Thermosonication in *B. bruxellensis* [73] and hypoxic growth conditions in *Cryptococcus neoformans* [74] have triggered this state. Moreover, Wang et al. [24] showed that cell-free *S. cerevisiae* supernatant produced the loss of culturability of *Hanseniaspora uvarum*, *M. pulcherrima* and *T. delbrueckii*, suggesting that the presence of some antimicrobial metabolites could induce the VBNC state. More specifically, Branco et al. [26] showed how antimicrobial peptides produced by *S. cerevisiae* caused the loss of culturability in *H. uvarum.* Regarding *S. cerevisiae*, for the first time, our study evidenced the presence of the VBNC state in mixed fermentation. This VBNC population was found in a high proportion (more than 85%) from the middle to the end of fermentation, reaching 93.74 ± 10.07%. Previous studies have demonstrated that *S. cerevisiae* is able to enter into a VBNC state under SO_2_ and heat shock stress [75,76]. In addition, Petitgonnet et al. [77] demonstrated that cell-to-cell contact between *S. cerevisiae* and *L. thermotolerans* modifies yeast metabolism and the exometabolome, and cell contact can also influence *S. cerevisiae* viability. In fact, common winemaking procedures include the use of SO_2_ to prevent the growth of unwanted microorganisms, among them, non-*Saccharomyces* yeasts. Industrial wine *S. cerevisiae* yeasts are known to present resistance to SO_2_, and this is one of the mechanisms of inducing VBNC status in non-*Saccharomyces* yeasts [23] and inducing the predominance of *S. cerevisiae* during the winemaking process. For this reason, in the present work, SO_2_ was not used to prevent the bias that would be introduced by its addition. Furthermore, the use of SO_2_ in the wine industry has been challenged due to side effects in human health; thus, our conditions are also useful in this new SO_2_-free scenario.

## 5. Conclusions

In summary, the use of the viable dye PMAxx coupled with qPCR is a reliable, specific and fast method for monitoring population dynamics in mixed fermentations. In addition to detecting minority yeasts until the end of fermentation, the presence of a VBNC state in *S. cerevisiae* was revealed for the first time during mixed alcoholic fermentation. Further research is still required to understand how the interactions between *S. cerevisiae* and non*-Saccharomyces* yeasts impact their physiological and metabolic status and which conditions produced during fermentation are causing this state.

## Figures and Tables

**Table 1:** Effect of PMAxx treatment in 10^6^ living or dead cells/mL of *Saccharomyces cerevisiae* (Sc), *Torulaspora delbrueckii* (Td), *Lachancea thermotolerans* (Lt) and *Metschnikowia pulcherrima* (Mp). (a) Ct reduction was obtained by subtracting the mean Ct values obtained from PMAxx treatment of living or dead cells from the mean Ct values obtained from untreated cells. (b) Ct reduction was obtained by subtracting the mean Ct values of dead cells from those of living cells, with or without PMAxx treatment. The results are expressed as the mean ± SD. The significance level for the unpaired *t*-test was *p* < 0.05.

	(a) ΔCt (PMAxx Treated–Untreated Cells)			(b) ΔCt (Dead–Living Cells)		
	Living Cells	Dead Cells	*p* Value	PMAxx	non-PMAxx	*p* Value
*S. cerevisiae*	2.003 ± 0.428	10.4 ± 1.558	0.001	15.42 ± 0.888	7.76 ± 1.447	0.018
*T. delbrueckii*	1.631 ± 1.040	5.566 ± 1.498	0.029	11.33 ± 1.736	7.392 ± 1.099	0.020
*L. thermotolerans*	2.171 ± 0.395	8.22 ± 0.310	0.010	13.46 ± 1.708	8.814 ± 0.3917	0.003
*M. pulcherrima*	0.5525 ± 0.665	4.834 ± 0.984	0.041	11.83 ± 1.170	7.548 ± 0.4837	0.036

**Table 2:** Slopes, Y-intersections, correlation coefficients (*R*^2^), efficiencies (%), standard errors, limits of quantification (LoQs) and limits of detection (LoDs) of standard curves obtained from serially diluted DNA of *S. cerevisiae*, *T. delbrueckii*, *L. thermotolerans*, *M. pulcherrima* and total yeasts, with or without the PMAxx dye. Efficiency (E) was calculated using the formula E = (10^−1/slope^)-1. ND, not determined.

	qPCR						
	Slope	Y-Intersection	*R* ^2^	Efficiency (%)	Error	LoQ	LoD
*S. cerevisiae*	−3.275	37.526	0.999	102	0.041	10^2^	10
*T. delbrueckii*	−3.149	37.174	1	107.77	0.027	10^2^	10
*L. thermotolerans*	−3.226	38.466	1	104.17	0.02	10^3^	10^2^
*M. pulcherrima*	−3.389	44.651	0.996	97.287	0.071	10^4^	10^3^
Total yeast	−3.539	40.168	0.995	91.657	0.085	ND	ND
	**PMAxx-qPCR**						
	**Slope**	**Y-Intersection**	***R*^2^**	**Efficiency (%)**	**Error**	**LoQ**	**LoD**
*S. cerevisiae*	−3.353	38.009	0.999	98.719	0.011	10^3^	10^2^
*T. delbrueckii*	−3.542	38.1	0.998	91.552	0.011	10^3^	10^2^
*L. thermotolerans*	−3.24	39.945	0.998	103.5	0.015	10^3^	10^2^
*M. pulcherrima*	−3.34	41.802	0.999	99.214	0.023	10^4^	10^3^
Total yeast	−3.129	37.672	0.994	108.73	0.074	ND	ND

## Appendix A

**Table A1:** Primer sequences used for quantitative PCR analysis.

Target Species	Primer Name	Sequence 5′-3′	Reference
*S. cerevisiae*	CESP-F	ATCGAATTTTTGAACGCACATTG	Hierro et al. [61]
	SCER-R	CGCAGAGAAACCTCTCTTTGGA	
*T. delbrueckii*	Tods L2	CAAAGTCATCCAAGCCAGC	García et al. [36]
	Tods R2	TTCTCAAACAATCATGTTTGGTAG	
*L. thermotolerans*	LTH2-F	CGCTCCTTGTGGGTGGGGAT	García et al. [36]
	LTH2-R	CTGGGCTATAACGCTTCTCC	
*M. pulcherrima*	MP2-F	AGACACTTAACTGGGCCAGC	García et al. [36]
	MP2-R	GGGGTGGTGTGGAAGTAAGG	
Total yeast	YEAST-F	GAGTCGAGTTGTTTGGGAATGC	Hierro et al. [34]
	YEAST-R	TCTCTTTCCAAAGTTCTTTTCATCTTT	

**Table A2:** Limit of Quantification (LoQ) and Limit of Detection (LoD) of *S. cerevisiae* (Sc), *T. delbrueckii* (Td), *L. thermotolerans* (Lt) and *M. pulcherrima* (Mp) obtained by PMAxx-qPCR. DNA from living cells of each species (Sc, Td, Lt or Mp) was mixed with DNA from dead cells of the same species (living/dead mixed cultures), or with DNA from living cells of another yeast species (living/living mixed cultures, Sc/Td, Td/Sc, Lt/Sc or Mp/Sc). Cycle threshold was calculated by triplicate and standard deviation was calculated (Ct ± SD).

Living/Dead Mixed Cultures			
Living Yeast (evaluated concentrations)	Dead Yeast	LoQ (Ct ± SD)	LoD (Ct ± SD)
Sc (10^4^ to 10 CFU/mL)	Sc (10^6^ CFU/mL)	10^3^ (27.907 ± 0.124)	10^2^ (30.841 ± 0.468)
Td (10^4^ to 10 CFU/mL)	Td (10^6^ CFU/mL)	10^3^ (28.670 ± 0.139)	10^2^ (30.817 ± 0.273)
Lt (10^4^ to 10 CFU/mL)	Lt (10^6^ CFU/mL)	10^3^ (29.594 ± 0.180)	10^2^ (32.255 ± 0.483)
Mp (10^4^ to 10 CFU/mL)	Mp (10^6^ CFU/mL)	10^4^ (28.570 ± 0.190)	10^3^ (32.030 ± 0.940)
**Living/Living Mixed Cultures**			
Living Yeast (evaluated concentrations)	Living Yeast	LoQ (Ct ± SD)	LoD (Ct ± SD)
Sc (10^4^ to 10 CFU/mL)	Td (10^7^ CFUs/mL)	10^3^ (28.057 ± 0.089)	10^2^ (30.992 ± 0.207)
Td (10^4^ to 10 CFU/mL)	Sc (10^7^ CFUs/mL)	10^3^ (29.922 ± 0.387)	10^2^ (32.121 ± 0.185)
Lt (10^4^ to 10 CFU/mL)	Sc (10^7^ CFUs/mL)	10^3^ (29.692 ± 0.280)	10^2^ (31.995 ± 0.759)
Mp (10^4^ to 10 CFU/mL)	Sc (10^7^ CFUs/mL)	10^4^ (29.597 ± 0.495)	10^3^ (31.173 ± 1.799)
